# Responses to social defeat in early- vs late-onset suicidal behavior: An experimental behavioral study

**DOI:** 10.1016/j.comppsych.2025.152657

**Published:** 2025-12-26

**Authors:** Anna Szücs, Elizabeth Campbell, Katalin Szanto, Alexandre Y. Dombrovski

**Affiliations:** a Faculty of Behavioural and Movement Sciences, Vrije Universiteit Amsterdam, Netherlands; b Department of Psychiatry, University of Pittsburgh, USA

**Keywords:** suicide, competition, social interactions, loss of status, interpersonal conflict, defeat, old age, narcissism

## Abstract

**Background:**

Social defeat is often cited as a motive for suicide. The experience of defeat may arise from feeling dominated in a dyadic conflict or from losing status in a group. We hypothesize that sensitivity to dyadic defeat will be related to the onset of suicidal behavior in early or mid-life and sensitivity to loss of status, in late life.

**Methods:**

The study's sample of 245 adults aged 50 years and older (mean = 63.2 years, SD = 7.4) comprised 42 early-onset and 32 late-onset suicide attempters (aged < 50 vs ≥ 50 years at their first suicide attempt), 114 depressed non-attempter comparisons, and 57 non-psychiatric comparisons. Using a validated rigged video game tournament task, we operationalized compensatory responses to the two forms of social defeat as point stealing from individual opponents (one-on-one defeat) and rank buying in the league table (loss of status in a group).

**Results:**

Early-onset attempters increased point stealing the most over time ($\chi_{3}^{2}=22.37$, *p* < .001), whereas late-onset attempters purchased more rank after losing trials than early-onset attempters and non-psychiatric comparisons ($\chi_{3}^{2}=9.47$, *p* = .024). Each effect was robust to adjusting for age and sex, other effects of interest, and to the exclusion of long-string responders.

**Conclusions:**

Our behavioral findings suggest that socio-behavioral processes leading to suicide vary across the life cycle. While vulnerability to dyadic defeat could be suicidogenic for people of any age, loss of social status could play a role in suicidal crises specifically occurring in later life.

## 1. Introduction

Clinical theorists argue that narcissistic concerns about self and status can trigger a suicidal crisis when an aging person cannot cope with loss of function and social standing [1–3], while empirical evidence for this is limited [4]. Older suicide decedents have been described as having self-oriented concerns about decline and loss of autonomy leading up to their suicidal act [5] and depressed older males cited such concerns as a motivation to contemplate or attempt suicide [6,7].

Among aging-related losses, older people at increased suicide risk may be particularly sensitive to loss of social status [8]. At the same time, less serious suicidal behavior in old age has been associated with interpersonal difficulties [9], serious suicidal behavior predicted retaliation in response to unfair offers in a bargaining task [10], and death by suicide in older men has been linked to marital conflicts and being cheated on by their partner [11].

These findings map onto the concept of social defeat, as described in the social defeat hypothesis of psychopathology [12,13]. This frame-work defines social defeat as either the failure to attain valued social or material resources (e.g., low social status) or exposure to interpersonal aggression and devaluation (e.g., being dominated in dyadic conflicts, humiliation) [12,13]. Alongside internal forms of defeat, such as self-criticism, social defeat experiences have been linked to a wide range of psychopathological outcomes, including suicide risk [13]. Defeat through loss or humiliation is also a core component of the Integrated Motivational–Volitional Model of suicidal behavior [14], further supporting its central role in late-life suicide. However, studies building on these theories have not investigated whether either form of social defeat, loss of status or interpersonal conflict, is a more prominent precipitant in old age than in younger age [13,15].

An aging-specific pathway to suicidal behavior should primarily operate in cases where the first suicidal crisis arises in late life, and older suicidal people with a first suicide attempt earlier in life provide a natural comparison group. As noted earlier, clinical theory holds that loss of status, common during aging [16], plays a relatively greater role in late-life suicide than interpersonal conflicts. Consistently, interpersonal conflicts are less common in older people who died in their first suicide attempt than in those with prior attempts [17]. Older depressed individuals with earlier-onset suicidal behavior display more Cluster B personality traits [18], and are more likely to generate stressors and experience interpersonal conflicts [19]. These findings suggest that early-onset attempters have a lifelong sensitivity to interpersonal forms of social defeat, whereas late-onset attempters are more likely to experience aging-related sensitivity to loss of status.

Behavioral processes involving distinct forms of social defeat have not yet been studied experimentally in older suicide attempters. Extant studies were observational, unable to tease out specific associations between suicidal behavior and loss of status vs one-on-one defeat, as they considered ambiguous social defeat outcomes, such as loss of employment [20–22], or failed to adjust for other social outcomes when investigating loss of status [6,8].

**Fig. 1.**
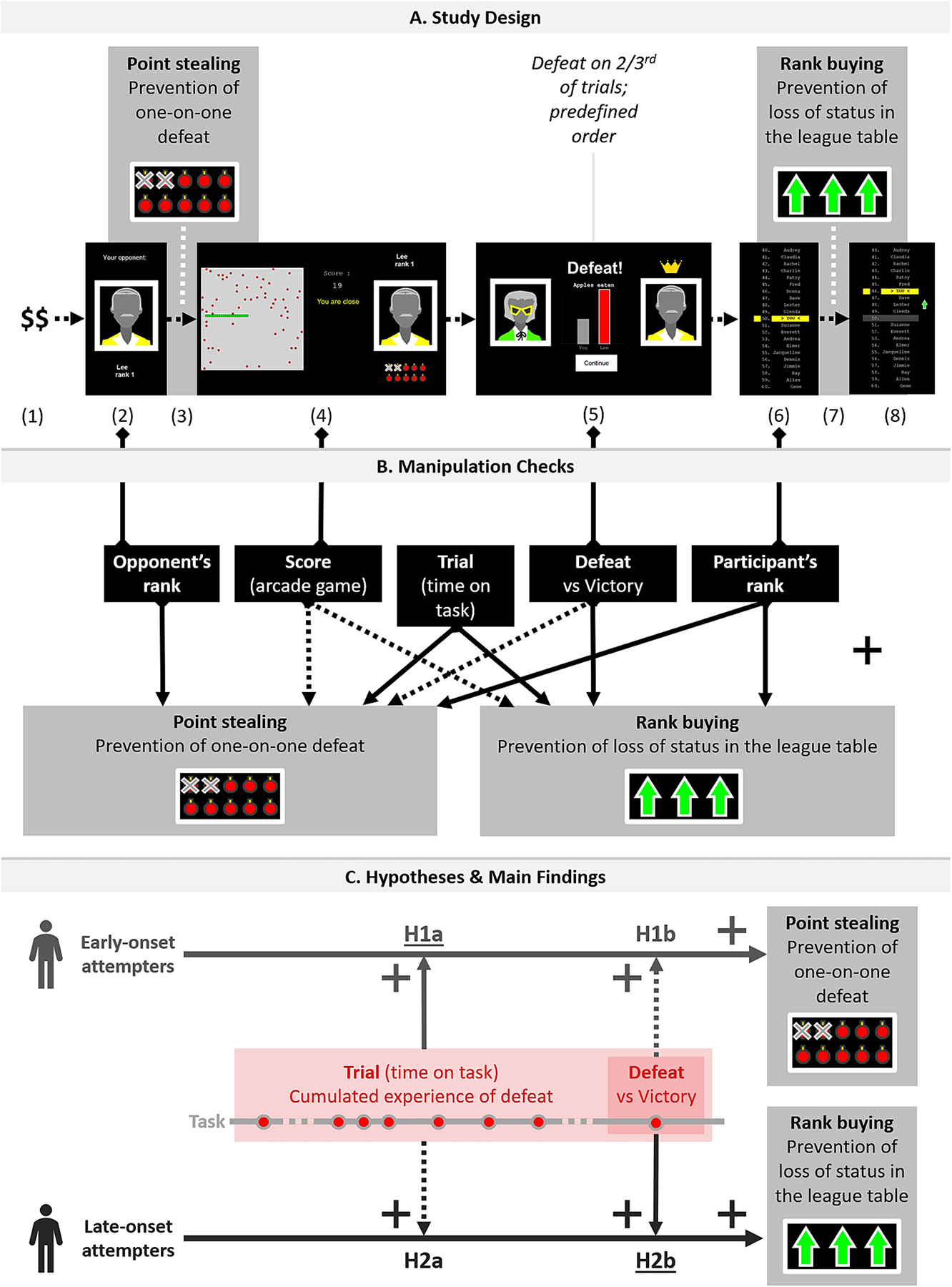
Pictorial summary of study design (Panel A), manipulation checks (Panel B), and hypothesis and main findings (Panel C). Note. Panel A – Sequence of events and actions during each trial of the CoBRA task: (1) Participants receive $20 virtual endowment they are free to spend on boosting their rank at the end of the trial; (2) participants get information about their next opponent (avatar, pseudonym, and rank in the league table); (3) participants can choose to steal points from the opponent before playing (point stealing outcome measure; see main text); (4) participants play a twenty-second session of the snake arcade game; (5) mock bar charts of participants' and their opponent's scores indicate whether they lost or won accompanied by a winning or losing jingle; (6) participants see their current rank in the league table, which increases by five ranks after winning trials and decreases by five ranks after losing trials; (7) participants can then choose whether they want to boost their rank by paying a portion of their virtual endowment received on the same trial (rank buying outcome measure; see main text); (8) participants see their final rank before starting the next trial. Figure adapted from its original published form [23]. Panel B – Manipulation checks: plain arrows indicate positive effects and dotted arrows indicate expected effects that were non-significant (see Supplement p. 1 for further details). Panel C – Hypotheses and main findings: Plain arrows indicate confirmed hypotheses and dotted arrows indicate unconfirmed ones.

Experiments can investigate sensitivity to different forms of social defeat more specifically. We have validated an experimental task capable of capturing behavioral reactions to one-on-one (interpersonal) defeat and overall loss of status separately: the Competitive Behavioral assessment of Rivalry and Admiration-seeking (CoBRA) task [23,24]. The behavioral outcomes measured by the task –behavioral rivalry, i.e., point stealing, and behavioral admiration-seeking, i.e., rank buying–reflect narcissistic responses to different ego threats, namely being dominated by others and loss of status [25]. In the CoBRA task (Fig. 1, Panels A and B), participants enter a rigged video game competition where they lose two-thirds of the time and can respond by stealing points from opponents before playing against them (rivalry) or by buying additional ranks in the tournament's league table after seeing their current rank (admiration-seeking). Both behaviors tend to increase as defeats accumulate during the task [23].

The CoBRA task has proven a valid simulation of real-life competitive contexts, able to capture affective and behavioral patterns conceptually defined as narcissism. First, across samples of different ages and levels of psychopathology, rivalry and admiration-seeking behaviors on the CoBRA task displayed replicable amplifications by self-reported trait narcissism [23]. Second, rivalry and admiration-seeking behaviors mapped specifically onto self-reported traits of narcissistic rivalry and admiration-seeking [24]. Third, grandiose and vulnerable narcissistic affects were found to vary similarly within the same individuals during the CoBRA task as during comparable real-life contexts measured by ecological momentary assessment [26]. Finally, trait narcissistic agency exacerbated the decrease in grandiose narcissistic affects in response to defeat in the CoBRA task [26].

In the present study, we use the behavioral measures of the CoBRA task to capture differential reactions to one-on-one defeat vs loss of status in people with early-onset vs. late-onset suicidal behavior in adults aged 50 years and older oversampled for depression and suicidal behavior. We have the following hypotheses (Fig. 1, Panel C):

[H1] Compared to late-onset attempters and non-attempter depressed and non-psychiatric comparisons, early-onset attempters will try to avert anticipated one-on-one (interpersonal) defeats more vigorously by stealing more points [H1a] as defeats accumulate over time, and [H1b] immediately following defeat trials;

[H2] compared to early-onset attempters and non-attempter depressed and non-psychiatric comparisons, late-onset attempters will buy increasingly more rank to avert loss of status in the group [H2a] as defeats accumulate over time, and [H2b] particularly following defeat trials.

We describe the associations of these behaviors with self-report measures of narcissism separately [27], but also report on narcissistic traits here to characterize the groups.

## 2. Methods

### 2.1. Participants

The sample consisted of 245 adults aged 50 years and older (mean age = 63.2 years, SD = 7.4, range = 50–85 years; with *n* = 138, 56.3 % females) who participated in the ongoing Longitudinal Research Program in Late-life Suicide in Pittsburgh, United States [9]. Three groups of participants were recruited: depressed non-attempter comparisons (*n* = 114), non-psychiatric comparisons (*n* = 57), and suicide attempters (*n* = 74). We classified suicide attempters into early- (*n* = 42) and late-onset (*n* = 32) attempter groups using a cut-off at < or ≥ 50 years of age at their first lifetime suicide attempt. This was in line with our previous work on personality and age of onset of suicidal behavior [18] and with common cutoffs for early- vs late-onset depression [28–30], on which the conceptualization of early- vs late-onset suicidal behavior was initially based [31]. We tested alternative cutoffs, which could have been equally justifiable, in sensitivity analyses (see subsection 2.5. Statistical Analysis).

Both early- and late-onset attempters had to have suicidal ideation in the month prior to enrollment and had made at least one suicide attempt during their life. Depressed non-attempters had to have clinical levels of depression at time of consent, as indicated by a score of at least 14 points on the 17-item Hamilton Rating Scale for Depression (HRSD) [32] and/or a current depressive disorder diagnosis based on Diagnostic and Statistical Manual of Mental Disorders 4th edition (DSM-IV) criteria as assessed by the Structured Clinical Interview for DFM (SCID) [33]. Non-psychiatric controls had no lifetime history of suicide attempt or ideation and no psychiatric illness. For all groups, exclusion criteria were the following: any SCID/DSM-IV diagnosis of bipolar disorder, psychosis, clinical diagnosis of dementia, intellectual disability, a Mini-Mental State Examination (MMSE) [34] score below 22, electroconvulsive therapy in the past six months, or any neurological or major systemic illness directly affecting the brain.

### 2.2. Procedure

All procedures were reviewed and approved by the Institutional Review Board at the University of Pittsburgh (STUDY19060351). Prior to participating in the experiment, all individuals provided written, informed consent. Data collection took place between September 2017 and March 2024, with a considerable slowdown between 2020 and 2022 caused by the COVID-19 Pandemic.

Participants played the CoBRA task on Windows tablets (see subsection 2.3. Behavioral Task). A research assistant walked them through the task instructions and a practice round. The participants then completed the task for 24 trials without interruption, which took approximately 20 min. The research assistant only observed participants' actions during the first two trials.

To preserve the integrity of the deception at the sample level given that some participants were acquainted or related, the rigged outcomes were not disclosed during data collection. However, the research assistant assessed participants' emotional state following task completion and conducted a thorough debriefing to monitor for any signs of distress related to the experiment. Any such concerns were promptly addressed by a psychiatrist on the study team.

Participants completed all other assessments reported below within the month of completing the CoBRA task (see subsection 2.4. Other Assessments).

### 2.3. Behavioral task

We employed the CoBRA task to investigate behavioral reactions to social defeat in a rigged video game tournament. This task is available on GitHub (https://github.com/aszucs/cobra_task_v1) and has been described in our previous work [23]. It is based on the classic *Snake* arcade game where players guide a snake to collect apples. The task consists in 24 trials (tournament rounds), each time divided into a contest phase (competing against different opponents) and a ranking phase (adjusting league standings based on the trial's outcome). See Fig. 1 (Panel A) for a detailed description.

To emulate a more realistic social environment, participants are led to believe that opponents are former study participants whose performance had been recorded. In reality, participants play against the same 24 virtual opponents in the same order, and win/lose in a similarly predefined sequence with a 2:1 defeat-to-victory ratio. On losing trials, participants lose against their opponent during the contest phase and then lose five ranks during the ranking phase.

The task records two main behavioral outcome measures, which are expected to increase as defeats accumulate throughout the task [23]: point stealing – stealing points from one's upcoming opponent before playing the snake arcade game (ranging from 1: no point stealing to 5: stealing 10 points/apples) and rank buying – purchasing extra ranks in the league table at the end of the trial (ranging from 1: no rank buying to 5: buying 5 ranks). Tournament rules highlight a cost to both behaviors: point stealing is meant to have a moral cost and is described as unsportsmanlike, whereas rank buying has a monetary cost, with instructions specifying that participants' real money reward will be based on how much virtual money they saved on average on three randomly selected trials. In reality, all participants received the same reward for undergoing the task, 25 USD.

### 2.4. Other assessments

Participants indicated their level of video game experience on a Likert scale at the beginning of the CoBRA task ranging from 1 – never played any games including on smartphones and tablets to 5 – playing every day.

To complement our behavioral assessment of dominance/defeat dynamics related to narcissism, the sample was characterized using the 60-item Five-Factor Narcissism Inventory–Short Form (FFNI-SF) [35]. The FFNI-SF contains self-report items scored using a five-point Likert scale ranging from 0 – Very untrue of me to 4 – Very true of me. In addition of total scores, we present scores for the narcissistic dimensions of agentic extraversion, antagonism, and narcissistic neuroticism. The FFNI-SF was available in 224 participants.

Depression severity was assessed by the 17-item HRSD, of which the item assessing suicide risk (Item 3) had been removed to prevent over-inflating depression severity scores in attempters. Without Item 3, HRSD scores range from zero to 50 points, with scores ≤ 7 points corresponding to no depression and ≥ 17 points to moderate to severe depression. The HRSD was clinician-administered within a week of task completion.

Suicidal ideation severity was assessed using the 19-item Scale for Suicidal Ideation (SSI) [36]. The SSI was clinician-administered within one month of completing the CoBRA task. Scores on the SSI range from 0 (no ideation or passive death wish) to 38 (maximum level of ideation). Participants' suicidal ideation over the past week (current ideation) and highest level of suicidal ideation experienced during their lifetime (worst ideation) were assessed.

Participants' suicidal behavior history was assessed by collecting their number of attempts and their age at their first attempt. Medical seriousness of their most medically serious attempt was assessed employing the eight-item Beck Lethality Scale (BLS) [37], a clinicianrated scale ranging from 0 – minimal medical seriousness to 10 – death. Suicidal intent and planning at participants' most medically serious attempts were additionally collected employing the clinicianrated, 20-item Beck Suicide Intent Scale (SIS) [38], and its eight-item planning subscale [39].

### 2.5. Statistical analysis

All analyses employed R, Version 4.2.2. We built separate sets of linear mixed-effects models predicting the two main outcome measures-point stealing and rank buying-with participant as a random effect; trial (time on task), opponent's rank, participant's current rank, and score on the snake arcade game as trial-level covariates; and age and sex entered in interaction with trial as participant-level covariates. All variance inflation factors were ascertained to be ≤ 3.

An initial analysis ensured that task dynamics replicated those reported in our previous publication on the same task [23] in the overall sample, and after excluding participants analyzed in our previous study.

We then built two sets of two main models to test H1 (increased point stealing in early-onset attempters vs other groups to prevent one-on-one defeat – Main Models 1a-b) and H2 (increased rank buying in late-onset attempters to prevent loss of status – Main Models 2a-b). Models 1a and 2a tested moderation of the effect of trial by study groups and Models 1b and 2b tested moderation of the effect of prior victory by study groups. Prior victory was coded 1 = victory, 0 = defeat; negative coefficients therefore indicated higher rank buying following defeat.

Finally, we conducted a series of sensitivity analyses to test the robustness of significant interaction effects in main models to: the presence of both tested interactions in a combined model (Sensitivity Analyses 1A and 2A); the inclusion of random slopes for the trial-level variable present in significant interactions (Sensitivity Analyses 1B and 2B); the omission of demographic covariates that had been included in main models, namely age and sex (Sensitivity Analyses 1C and 2C); and the exclusion of participants with low task engagement, as assessed by their unvaried point stealing and rank buying responses throughout the entire task (long-string responders; Sensitivity Analyses 1D and 2D). We additionally refit our main models with alternative cutoffs for early- vs late-onset attempters (Supplemental Tables S2 and S3), namely < 43 vs ≥ 43 years corresponding to a median split of age of onset of suicidal behavior, and < 60 years vs ≥ 60 years corresponding to the standard age cutoff for old age used by the World Health Organization [40] and also widely employed in studies on early- vs late-onset depression [30].

## 3. Results

### 3.1. Sample characteristics

Table 1 presents all sample characteristics. Of the 245 participants, 74 were suicide attempters (42 early-onset attempters; 32 late-onset attempters), 114 depressed non-attempters, and 57 non-psychiatric comparison participants (Table 1). The mean age was 63.2 years (SD = 7.4), 56.3 % participants were female at birth, and most were White (84.9 %). Early-onset attempters were more likely to be female than all other groups; our subsequent analyses controlled for sex.

Depressed groups (*n* = 188) had a mean score of 19.2 points (SD = 5.6) on the HRSD, corresponding to moderate to severe depression. Attempter groups were more severely depressed and had more intense current and worst lifetime suicidal ideation than depressed non-attempter comparisons.

As expected, early-onset attempters were younger at their first suicide attempt, which occurred at an average age of 25.8 years (SD = 12.4), as opposed to an average age of onset of 60.2 years (SD = 7.8) for late-onset attempters. Most early-onset attempters had attempted suicide multiple times (27 multiple vs 15 single attempts), whereas most late-onset attempters had made a single attempt (10 multiple vs 22 single attempts).

With respect to narcissistic traits, depressed groups displayed higher levels of narcissism than non-psychiatric comparisons, mostly driven by higher narcissistic neuroticism and antagonism. Additionally, early-onset attempters had higher levels of narcissistic neuroticism than late-onset attempters.

### 3.2. Manipulation checks

Behavioral responses to defeat in the overall current sample are summarized in Fig. 1 (Panel B) and described in detail in the Supplement (Supplement p. 1; Supplemental Table S1). Manipulation effects replicated those previously reported [23].

### 3.3. Group differences in point stealing (averting one-on-one defeat, H1)

**Fig. 2.**
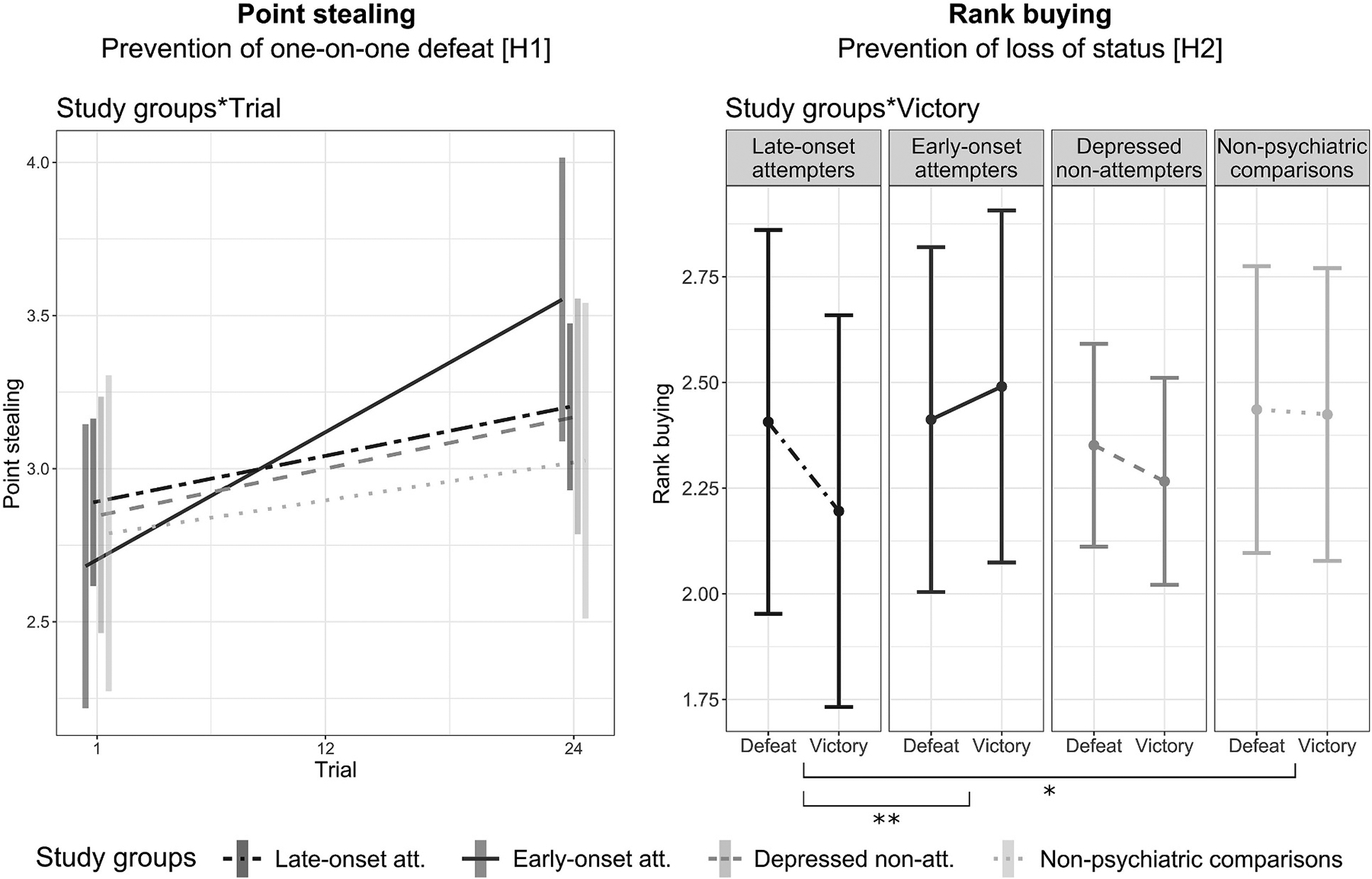
Moderating effects of study groups on point stealing (left) and rank buying (right). Note. Error bars represent standard errors. Legend: att., attempters; *, *p* < .05; **, *p* < .01.

Table 2 presents tests of H1 and Fig. 1 (Panel C) presents a summary of main findings. As defeats accumulated, point stealing increased (B = 0.25, SE = 0.03, β = 0.14, *p* < .001), with early-onset attempters stealing progressively more points than all other groups (Main model 1a: $\chi_{3}^{2}=22.37$, *p* < .001; Fig. 2 – left plot), namely late-onset attempters (B = −0.19, SE = 0.05, β = −0.11, *p* < .001), depressed non-attempter comparisons (B = −0.17, SE = 0.04, β = −0.09, *p* < .001), and non-psychiatric comparisons (B = −0.17, SE = 0.04, β = −0.09, *p* < .001). Although our previous larger study showed that defeats had to accumulate over several minutes to have an affective impact [23], we examined effects of single defeats as well. Point stealing following defeat on the previous trial did not differ from point stealing after victory on the previous trial nor was it moderated by study group. In summary, H1a was supported whereas H1b was not, with early-onset attempters engaging in more point stealing than other groups over time but with no group differences found in point stealing following defeat trials.

In sensitivity analyses, the group by trial interaction was robust to controlling for a group by prior defeat interaction (Sensitivity Analysis 1A; $\chi_{3}^{2}=21.94$, *p* < .001), to omitting participant-level covariates (Sensitivity Analysis 1C; $\chi_{3}^{2}=23.58$, *p* < .001), and to excluding long-string responders (Sensitivity Analysis 1D; $\chi_{3}^{2}=22.34$, *p* < .001). In Sensitivity Analysis 1B including random slopes for trial to account for random inter-individual heterogeneity, group differences retained their direction and magnitude compared to Main Model 1a, although only the difference between early-onset attempters and depressed non-attempters remained significant (B = −0.17, SE = 0.08, β = −0.09, *p* = .029). Alternative cutoffs for early- vs late-onset attempters, namely 43 years (median split) and 60 years (standard cutoff for old age) did not change main findings for point stealing (Supplemental Table S2).

### 3.4. Group differences in rank buying (averting loss of status, H2)

Table 3 presents tests of H2 and Fig. 1 (Panel C) presents a summary of main findings. We found no group differences in rank buying over time. However rank buying increased after defeat on the previous trial (B = −0.21, SE = 0.08, β = −0.13, *p* = .009) and was further moderated by study group (Main model 2b: $\chi_{3}^{2}=9.47$, *p* = .024; Fig. 2 – right plot), such that late-onset attempters bought rank preferentially after being defeated and losing rank in the league table, differing from early-onset attempters and non-psychiatric comparisons (resp. B = 0.29, SE = 0.10, β = 0.18, *p* = .004 and B = 0.20, SE = 0.09, β = 0.12, *p* = .031). In summary, H2a was not supported, whereas H2b was supported: although there were no group differences in rank buying over time, late-onset attempters engaged in more rank buying after defeat trials.

In sensitivity analyses, this group by prior defeat interaction was robust to controlling for the group by trial interaction (Sensitivity Analysis 2A; $\chi_{3}^{2}=9.60$, *p* = .022), to omitting participant-level covariates (Sensitivity Analysis 2C; $\chi_{3}^{2}=8.48$, *p* = .037), and to excluding long-string responders (Sensitivity Analysis 2D; $\chi_{3}^{2}=9.86$, *p* = .020). In Sensitivity Analysis 2B including random slopes for prior victory, group differences significant in Main Model 2b retained their direction and magnitude, but the only significant group difference was between late-and early-onset attempters (B = 0.29, SE = 0.12, β = 0.18, *p* = .017).

The difference between early-onset and late-onset attempters was also present with alternative age cutoffs, namely 43 years (median split) and 60 years (standard cutoff for old age), but the difference between late-onset attempters and non-psychiatric comparisons no longer reached significance with the 43-years cutoff (Supplemental Table S3). When using a 60-years cutoff, the difference in rank buying after losing trials became significant between late-onset attempters and all other groups (Supplemental Table S3).

## 4. Discussion

Using a rigged video game behavioral experiment, we tested whether two forms of social defeat, namely one-on-one defeat vs loss of status had a differential impact on people with early- vs. late-onset suicidal behavior. Consistently with our hypotheses, we found that early-onset suicide attempters tried to prevent one-on-one defeat particularly vigorously (supporting H1a), whereas late-onset attempters displayed an exaggerated immediate response to loss of status with more compensatory rank buying (supporting H2b). These group differences held with or without controlling for demographics and when accounting for inter-individual heterogeneity or excluding long-string responders. However, no group differences emerged in the prevention of one-on-one defeat immediately following defeat trials, nor in status-recovering behavior over time, thus providing no support for our alternative hypotheses H1b and H2a.

The focus of early-onset attempters on preventing anticipatory defeat is in line with our prior cross-sectional observations linking early-onset suicidal behavior to interpersonal difficulties [19]. Participants were told that point stealing was unsportsmanlike, but other players could also engage in this behavior. Averting defeat by such means may thus constitute an antagonistic or even antisocial strategy. Alternatively, excessive efforts to prevent defeat may represent a measure to avoid subordination that may lead to depression [41] or humiliation and feelings of entrapment [14], described as potential precipitants of suicide contemplation by dominance behavioral system and integrated motivational-volitional theories of suicidal behavior, respectively. Although early-onset attempters scored higher on narcissistic neuroticism than late-onset attempters, they also started the game with low levels of point stealing, suggesting reduced early engagement in competitive behaviors and progressive adaptation to the environment's level of hostility (i.e., to the accumulating experience of defeat). This pattern speaks against indiscriminate defeat avoidance, typically associated with narcissistic neuroticism [27,42]. Additionally, early-onset attempters engaged in relatively more rank buying after winning trials, which aligns better with dominant rather than defensive motivations and has been linked to more grandiose forms of narcissism both in the context of this task [24,27] and generally [25,43]. However, we previously found that early-onset attempters represent a heterogeneous group in terms of internalizing and externalizing traits, with only some having a grandiose narcissistic and antagonistic profile [44]. This suggests that the observed behavioral patterns may reflect a mix of aggressive and defeat-avoidance motivations. Regardless, these findings support that early-onset attempters may display a lifelong pattern of excessive response to interpersonal difficulties. A specific association of dyadic defeat and vulnerability to suicidal behavior in old age will nonetheless require prospective confirmation.

The urgent response of late-onset attempters to status threats reveal a more self-focused concern about perceived status as compared to early-onset attempters' outward-directed behavioral response to interpersonal defeat. This aligns with the idea that late-life suicide can result from aversion to aging-related losses [1]. Preoccupation with losses and regaining control are central to qualitative accounts of suicide and self-neglect in old age [45]. Late-onset attempters did not display higher levels of narcissistic traits than other depressed groups, consistently with our prior research rather characterizing their personality as orderly, with a high need for control [18,44]. Older suicide decedents, who tend to be mostly first-time attempters [46], have also been described as authoritarian and controlling [47]. Further, we found that higher levels of conscientiousness are associated with recent suicide attempts and greater suicidal intent in middle-aged and older adults [48]. Thus, the hypersensitivity to loss of status observed in late-onset attempters is more likely to correspond to attempts to regain control over the situation than to narcissistic self-enhancement, although motivational inferences can only be speculative. Consistently with this hypothesis, the short timescale of rank buying immediately after losing trials suggests a different behavioral dynamic than rank buying in response to the cumulated experience of defeat observed with narcissism [23]. Such a reaction may align better with a striving for control and achievement, as seen in more conscientious people more negatively affected by unemployment [49]. Above all, this finding highlights the clinical need to explore the meaning of losses as likely risk factors for suicidal behavior and to address the distress they cause in depressed older adults.

Strengths of the present study include a careful experimental assessment of behavioral reactions to social defeat, and a well-characterized clinical sample. Our experimental design enabled to distinguish two forms of social defeat, which is challenging in naturalistic studies. Although one-on-one defeat and loss of social status have been commonly distinguished in rodents [50,51], these two constructs have rarely been contrasted in humans, even in the laboratory [52].

A few limitations should also be mentioned. First, given the large number of interaction effects to be tested, our sample size may not have afforded sufficient power to detect more subtle group differences, particularly when including random slopes. Despite all sensitivity analyses, the risk of Type I and II errors remains non-negligible, in particular regarding differences between attempter subgroups, which had less than 50 participants each. Second, the task setup, while intended to simulate real-world social competition, was a short computer game with virtual opponents, which may not fully capture the complexity of social defeat experiences and could limit the generalizability of findings to natural social contexts. In particular, interactions with virtual opponents may have failed to evoke the same affective responses in participants as real-life encounters. In addition, given the task's complex demands, future studies will need to rule out competing explanations of the observed results. Finally, we did not test associations between task-related behavioral dynamics and prospective suicide risk. This precludes any causal inference between behaviors observed with the task and suicidal behavior. Accordingly, the motivational or trait-level interpretations of task-based behaviors discussed above remain hypothetical and require confirmation by future studies.

This experimental study constitutes a first attempt to understand behavioral pathways from perceived social defeat to suicidal behavior across the lifespan, suggesting that dyadic defeat could be suicidogenic in all ages, unlike status threats, which may become more perilous in late life. To refine our understanding of these behavioral dynamics further, the two outcome measures of the CoBRA task could be revised to separate aggression from defeat avoidance on the one hand, and self-enhancement from status control on the other. Exploratory approaches such as latent profile analysis or other algorithmic clustering methods can further help parse potential heterogeneity within attempter groups and clarify links between their traits, motivations, and behaviors on the task. By combining such task-based studies with longitudinal follow-up, we may map the behavioral and affective pathways to suicide, linking personality traits with trait-congruent stressors, which could help early prevention efforts and inform psychotherapeutic approaches. However, relevant early interventions can already start addressing the mechanisms outlined in this work. One potential approach involves prompt, targeted management of family conflict and interpersonal difficulties in aging patients with early-onset mental disorders or suicidal crises using treatments such as systemic or interpersonal therapy [53,54]. Another involves promoting community engagement through hobbies, volunteering, or other group activities in older adults experiencing aging-related losses to help diversify their sources of social connection and recognition [55,56].

## Supplementary Material

1

## Figures and Tables

**Table 1:** Sample characteristics.

	Total sample *N* = 245	loSA *n* = 32	eoSA *n* = 42	DNA *n* = 114	NPC *n* = 57	Test statistics, effect size	*p*-value	post-hoc pairwise tests
*Socio-demographic characteristics*								
Age in years	63.2 (7.4)	64.5 (8.2)	61.2 (7.1)	63.0 (7.0)	64.1 (7.7)	F(3,241) = 1.73, η^2^ = 0.02	.161	–
Female sex - count (%)	138 (56.3 %)	14 (43.8 %)	33 (78.6 %)	62 (54.4 %)	29 (50.9 %)	χ^2^(3) = 11.37, Cramer's V = 0.19	**.010**	others groups < eoSA
Race - count (%):						Fisher's exact test, Cramer's V = 0.00	.358	–
Black	30 (12.2 %)	4 (12.5 %)	6 (14.3 %)	16 (14.0 %)	4 (7.0 %)			
White	208 (84.9 %)	26 (81.3 %)	34 (81.0 %)	95 (83.3 %)	53 (93.0 %)			
other	7 (2.9 %)	2 (6.3 %)	2 (4.8 %)	3 (2.6 %)	0 (0.0 %)			
Game Experience	2.3 (1.5)	2.1 (1.3)	2.6 (1.6)	2.4 (1.5)	2.1 (1.2)	F(3,241) = 1.16, η^2^ = 0.01	.326	–
*Depression, suicidal ideation, and suicidal behavior*								
Current depression severity (HRSD)	19.2 (5.6)	20.8 (6.4)	20.9 (5.8)	18.1 (5.1)	–	F(2,185) = 5.74, η^2^ = 0.06	**.004**	DNA < eoSA, loSA
Current suicidal ideation severity (SSI)	6.4 (9.1)	11.2 (12.7)	10.6 (9.7)	3.5 (6.2)	–	F(2,185) = 17.33, η^2^ = 0.16	**<.001**	DNA < eoSA, loSA
Worst lifetime suicidal ideation severity (SSI)	9.7 (11.3)	15.4 (13.5)	15.7 (12.0)	5.8 (8.4)	–	F(2,185) = 20.24, η^2^ = 0.18	**<.001**	DNA < eoSA, loSA
Age of onset at first attempt	40.7 (20.2)	60.2 (7.8)	25.8 (12.4)	–	–	F(1,72) = 188, η^2^ = 0.72	**<.001**	eoSA < loSA
Number of attempts - median [IQR]	1.5 [1–3]	1 [1–2]	2 [1–4]	–	–	χ^2^(1) = 8.12, $\eta_{\text{H}}^{2}$ = 0.10	**.004**	loSA < eoSA
Worst lifetime suicide attempt lethality score (BLS)	3.4 (2.0)	3.7 (1.7)	3.1 (2.1)	–	–	F(1,72) = 2.02, η^2^ = 0.03	.160	–
Worst lifetime suicidal intent (SIS)	18.3 (5.4)	19.4 (5.9)	17.5 (4.9)	–	–	F(1,72) = 2.18, η^2^ = 0.03	.144	–
Worst lifetime suicidal planning (SIS subscore)	7.8 (3.1)	8.3 (3.7)	7.4 (2.5)	–	–	F(1,72) = 1.61, η^2^ = 0.02	.209	–
*Narcissistic personality traits*								
Narcissism (FFNI-SF)	2.3 (0.4)	2.3 (0.4)	2.4 (0.4)	2.3 (0.4)	2.1 (0.3)	F(3,220) = 5.12, η^2^ = 0.07	**.002**	NPC < other groups
Narcissistic Extraversion (FFNI-SF subscore)	2.7 (0.7)	2.8 (0.7)	2.6 (0.7)	2.7 (0.8)	2.8 (0.6)	F(3,220) = 1.01, η^2^ = 0.01	.387	–
Narcissistic Antagonism (FFNI-SF subscore)	1.9 (0.5)	2.0 (0.5)	2.0 (0.5)	1.9 (0.5)	1.6 (0.3)	F(3,220) = 6.62, η^2^ = 0.08	**<.001**	NPC < other groups
Narcissistic Neuroticism (FFNI-SF subscore)	3.0 (0.9)	2.9 (0.7)	3.5 (0.8)	3.2 (0.9)	2.4 (0.6)	F(3,220) = 17.49, η^2^ = 0.19	**<.001**	NPC < other groups; loSA < eoSA

Note. Numbers indicate mean (SD) unless otherwise specified. Significant *p*-values for group differences are bolded. Pairwise differences were obtained with Tukey's honestly significance difference. Of all measures, only narcissism scores were missing in 21 participants. Legend: eoSA, early-onset suicide attempters; loSA, late-onset suicide attempters; DNA, depressed non-attempters; NPC, non-psychiatric comparisons; HRSD, Hamilton Rating Scale for Depression; SSI, Beck Scale of Suicidal Ideation; BLS, Beck Lethality Scale; SIS, Beck Suicide Intent Scale; FFNI-SF, Five-Factor Narcissism Inventory–Short Form.

**Table 2:** Point stealing: manipulation effects and group differences, and controlling for age and sex.

Dependent variable:	Point stealing – prevention of one-on-one defeat [H1] B (standard error), standardized β					
	Main Model 1a (testing group differences in the effect of trial)	Main Model 1b (testing group differences in the effect of last trial's outcome)	Sensitivity Model 1A (combining Main Models 1 and 2)	Sensitivity Model 1B (including random slopes for Trial)	Sensitivity Model 1C (without participant-level covariates)	Sensitivity Model 1D (excluding 31 long-string responders)
*Trial-level (design) variables*						
Trial	0.25*** **(0.03), 0.14**	0.12*** **(0.01), 0.07**	0.25*** **(0.03), 0.14**	0.25** **(0.07), 0.14**	0.26*** **(0.03), 0.15**	0.28*** **(0.04), 0.16**
Victory (vs Defeat)	*−*0.01 (0.03), −0.01	−0.04 (0.07), −0.02	−0.02 (0.07), −0.01	−0.02 (0.03), −0.01	−0.02 (0.03), −0.01	−0.01 (0.03), −0.01
Opponent's rank	0.09*** **(0.01), 0.06**	0.09*** **(0.01), 0.06**	0.09*** **(0.01), 0.06**	0.09*** **(0.01), 0.06**	0.09*** **(0.01), 0.06**	0.11*** **(0.02), 0.07**
Score	−0.001 (0.02), −0.001	0.001 (0.02), 0.001	−0.001 (0.02), −0.001	−0.01 (0.01), −0.003	0.001 (0.02), 0.001	0.0003 (0.02), 0.0002
Current rank	0.05* **(0.02), 0.03**	0.06* **(0.02), 0.03**	0.05* **(0.02), 0.03**	0.09* **(0.04), 0.05**	0.06* **(0.02), 0.03**	**0.05 (0.03), 0.03**
*Study groups*						
Study group (ref: Early-onset attempters):						
** **Late-onset attempters	−0.21 (0.35), −0.13	−0.22 (0.35), −0.13	−0.20 (0.35), −0.12	−0.21 (0.34), −0.13	−0.25 (0.34), −0.16	−0.12 (0.35), −0.08
** **Depressed non-attempters	−0.07 (0.26), −0.05	−0.12 (0.27), −0.07	−0.10 (0.27), −0.07	−0.07 (0.26), −0.05	−0.10 (0.26), −0.06	−0.08 (0.26), −0.06
** **Non-psychiatric comparisons	−0.11 (0.30), −0.07	−0.12 (0.30), −0.07	−0.10 (0.30), −0.06	−0.11 (0.30), −0.07	−0.15 (0.29), −0.09	0.11 (0.30), 0.06
Study group*Trial (ref: Early-onset attempters*Trial):						
** **Late-onset attempters*Trial	−0.19*** **(0.05), −0.11**	–	−0.19*** **(0.05), −0.11**	−0.19 (0.10), −0.10	−0.19*** **(0.05), −0.11**	−0.22*** **(0.06), −0.13**
** **Depressed non-attempters*Trial	−0.17*** **(0.04), −0.09**	–	−0.17*** **(0.04), −0.09**	−0.17* **(0.08), −0.09**	−0.17*** **(0.04), −0.09**	−0.19*** **(0.05), −0.11**
** **Non-psychiatric comparisons*Trial	−0.17*** **(0.04), −0.09**	–	−0.17*** **(0.04), −0.09**	−0.17 (0.09), −0.09	−0.17*** **(0.04), −0.09**	−0.19*** **(0.04), −0.11**
Study group*Victory (ref: Early-onset attempters*Victory):						
** **Late-onset attempters*Victory	–	0.01 (0.10), 0.003	−0.02 (0.10), −0.01	–	–	–
** **Depressed non-attempters*Victory	–	0.11 (0.08), 0.07	0.09 (0.08), 0.05	–	–	–
** **Non-psychiatric comparisons*Victory	–	0.01 (0.09), 0.003	−0.02 (0.09), −0.01	–	–	–
*Participant-level covariates*						
Male sex (vs female)	0.01 (0.19), 0.01	0.03 (0.19), 0.02	0.03 (0.19), 0.02	0.004 (0.19), 0.003	–	0.14 (0.19), 0.08
Age	−0.11 (0.09), −0.06	−0.12 (0.09), −0.07	−0.12 (0.09), −0.07	−0.11 (0.09), −0.06	–	−0.13 (0.09), −0.08
Male sex*Trial (vs female sex*Trial)	0.03 (0.03), 0.01	–	0.02 (0.03), 0.01	0.03 (0.06), 0.01	–	0.04 (0.03), 0.02
Age*Trial	−0.02 (0.01), −0.01	–	−0.02 (0.01), −0.01	−0.02 (0.03), −0.01	–	−0.02 (0.02), −0.01
Male sex*Victory (vs female sex*Victory)	–	−0.07 (0.05), −0.04	−0.07 (0.05), −0.04	–	–	–
Age*Victory	–	0.03 (0.03), 0.02	0.03 (0.03), 0.02	–	–	–
*Constant*	3.12*** **(0.23)**	3.13*** **(0.23)**	3.12*** **(0.23)**	3.12*** **(0.23)**	3.15*** **(0.22)**	3.17*** **(0.23)**

Note. All continuous predictors were mean-centered and scaled. For each data point of the dependent variable, models used the most recent preceding data point for all trial-level predictors (e.g., *Score* refers to the participants' score on the snake arcade game of the previous trial). Significant effects are bolded.

Legend:

*, p < .05;

**, p < .01;

***, *p* < .001.

**Table 3:** Rank buying: manipulation effects and group differences, controlling for age and sex.

Dependent variable:	Rank buying – prevention of loss of status [H2] B (standard error), standardized β					
	Main Model 2a (testing group differences in the effect of trial)	Main Model 2b (testing group differences in the effect of last trial's outcome)	Sensitivity Model 2A (combining Main Models 1 and 2)	Sensitivity Model 2B (including random slopes for Victory)	Sensitivity Model 2C (without participant-level covariates)	Sensitivity Model 2D (excluding 31 long-string responders)
*Trial-level (design) variables*						
Trial	0.05 (0.04), 0.03	0.07*** **(0.01), 0.04**	0.05 (0.04), 0.03	0.07*** **(0.01), 0.04**	0.07*** **(0.01), 0.04**	0.07*** **(0.02), 0.04**
Victory (vs Defeat)	−0.06* **(0.03), −0.03**	−0.21** **(0.08), −0.13**	−0.21** **(0.08), −0.13**	−0.22* **(0.10), −0.13**	−0.20** **(0.07), −0.12**	−0.25** **(0.09), −0.15**
Opponent's rank	0.03 (0.01), 0.02	0.03 (0.01), 0.02	0.03 (0.01), 0.02	0.03 (0.01), 0.02	0.03 (0.01), 0.02	0.03 (0.02), 0.02
Score	0.001 (0.01), 0.001	0.002 (0.01), 0.001	0.001 (0.01), 0.001	0.001 (0.01), 0.001	0.003 (0.01), 0.002	0.005 (0.02), 0.003
Current rank	0.07** **(0.02), 0.05**	0.08*** **(0.02), 0.05**	0.07** **(0.02), 0.05**	0.07** **(0.02), 0.05**	0.08*** **(0.02), 0.05**	0.08** **(0.03), 0.05**
Within-person mean rank buying	0.18*** **(0.01), 0.11**	0.18*** **(0.01), 0.11**	0.18*** **(0.01), 0.11**	0.19*** **(0.01), 0.12**	0.18*** **(0.01), 0.11**	0.19*** **(0.01), 0.12**
*Study groups*						
Study group (ref: Late-onset attempters):						
Early-onset attempters	0.09 (0.31), 0.06	0.01 (0.31), 0.003	0.003 (0.31), 0.005	0.005 (0.31), 0.003	−0.04 (0.31), −0.02	−0.05 (0.33), −0.03
Depressed non-attempters	−0.02 (0.26), −0.01	−0.06 (0.26), −0.03	−0.05 (0.26), −0.03	−0.06 (0.26), −0.04	−0.07 (0.26), −0.04	−0.12 (0.27), −0.07
Non-psychiatric comparisons	0.09 (0.29), 0.06	0.03 (0.29), 0.02	0.03 (0.29), 0.02	0.03 (0.29), 0.02	0.02 (0.29), 0.01	0.11 (0.30), 0.07
Study group*Trial (ref: Late-onset attempters*Trial):						
Early-onset attempters*Trial	0.06 (0.05), 0.04	–	0.06 (0.05), 0.04	–	–	–
Depressed non-attempters*Trial	−0.01 (0.04), −0.004	–	−0.01 (0.04), −0.003	–	–	–
Non-psychiatric comparisons*Trial	0.03 (0.04), 0.02	–	0.03 (0.04), 0.02	–	–	–
Study group*Victory (ref: Late-onset attempters*Victory):						
Early-onset attempters*Victory	–	0.29** **(0.10), 0.18**	0.29** **(0.10), 0.18**	0.29* **(0.12), 0.18**	0.26** **(0.10), 0.16**	0.34** **(0.12), 0.21**
Depressed non-attempters*Victory	–	0.13 (0.08), 0.08	0.13 (0.08), 0.08	0.13 (0.10), 0.08	0.11 (0.08), 0.07	0.15 (0.10), 0.09
Non-psychiatric comparisons*Victory	–	0.20* **(0.09), 0.12**	0.20* **(0.09), 0.12**	0.20 (0.11), 0.13	0.20* **(0.09), 0.12**	0.23* **(0.11), 0.14**
*Participant-level covariates*						
Male sex (vs female)	0.24 (0.17), 0.15	0.24 (0.17), 0.15	0.24 (0.17), 0.15	0.24 (0.17), 0.15	–	0.25 (0.18), 0.15
Age	−0.06 (0.08), −0.04	−0.08 (0.08), −0.05	−0.08 (0.08), −0.05	−0.08 (0.08), −0.05	−	−0.09 (0.09), −0.06
Male sex*trial (vs female sex*Trial)	0.01 (0.03), 0.01	–	0.01 (0.03), 0.01	–	–	–
Age*Trial	−0.005 (0.01), −0.003	–	−0.005 (0.01), −0.003	–	–	–
Male sex*Victory (vs female sex*Victory)	–	0.003 (0.05), 0.002	0.004 (0.05), 0.002	0.01 (0.07), 0.003	–	0.005 (0.06), 0.003
Age*Victory	–	0.06* **(0.03), 0.04**	0.06* **(0.03), 0.04**	0.06 (0.03), 0.04	–	0.07* **(0.03), 0.04**
*Constant*	2.24*** **(0.25)**	2.28*** **(0.25)**	2.28*** **(0.25)**	2.28*** **(0.25)**	2.40*** **(0.23)**	2.43*** **(0.26)**

Note. All continuous predictors were mean centered and scaled. For each data point of the dependent variable, models used the most recent preceding data point for all trial-level independent variables (e.g., *Score* refers to the participants' score of the same trial). *Within-person mean rank* was included in the models to account for the increase in rank resulting from previous rank buying choices. Significant effects are bolded.

Legend:

*, p < .05;

**, p < .01;

***, p < .001.

## Data Availability

Data is available from the corresponding author upon reasonable request.

## Appendix A. Supplementary data

Supplementary data to this article can be found online at https://doi.org/10.1016/j.comppsych.2025.152657.
