# Supplementary material for: Responses to social defeat in early- vs late-onset suicidal behavior: An experimental behavioral study
Source: Compr Psychiatry. Author manuscript; Available in PMC 2026 Jul 7. (PMC13339945; doi:10.1016/j.comppsych.2025.152657)
Supplement: 1 [file NIHMS2188777-supplement-1.docx]

**Supplement to the research article titled “Responses to social defeat in early- vs late-onset suicidal behavior: an experimental behavioral study”**

**Task dynamics, replication**

Supplementary Table S1 displays all replication models of task dynamics. As in our initial study (Szücs, Szanto, Adalbert, et al., 2020), both point stealing and rank buying increased with trial (time on task; for point stealing: B = 0.12, SE = 0.02, β = 0.07, p <.001; for rank buying: B = 0.06, SE = 0.02, β = 0.04, p < .001), and point stealing also increased with higher opponent’s rank (B = 0.09, SE = 0.01, β = 0.06, p <.001).

Additional main effects not found in our previously published analysis included both point stealing and rank buying increasing with higher rank in the tournament’s league table (for point stealing: B = 0.06, SE = 0.02, β = 0.03, p = .015; for rank buying: B = 0.08, SE = 0.02, β = 0.05, p < .001) and rank buying decreasing with prior victory on the snake arcade game (B = -0.06, SE = 0.03, β = -0.03, p = .045).

**Supplementary Table S1**

*Summary table of linear mixed-effects models predicting point stealing and rank buying, testing task dynamics, and controlling for age and sex in the overall sample (N = 245).*

| Dependent variable: | **Point stealing**  B (standard error), standardized β | **Rank buying**  B (standard error), standardized β |
| --- | --- | --- |
| Trial | **0.12^***^ (0.02), 0.07** | **0.06^***^ (0.02), 0.04** |
| Victory (vs Defeat) | -0.02 (0.03), -0.01 | **-0.06^*^ (0.03), -0.03** |
| Opponent’s rank | **0.09^***^ (0.01), 0.06** | 0.03 (0.01), 0.02 |
| Score | -0.0001 (0.02), -0.00002 | 0.002 (0.01), 0.001 |
| Current rank | **0.06^*^ (0.02), 0.03** | **0.08^***^ (0.02), 0.05** |
| Within-person mean rank | - | **0.18^***^ (0.01), 0.11** |
| Male sex (vs female) | -0.01 (0.18), -0.01 | 0.23 (0.17), 0.14 |
| Age | -0.11 (0.09), -0.07 | -0.06 (0.08), -0.04 |
| Trial*Male sex (vs trial*female sex) | 0.001 (0.03), 0.0004 | 0.01 (0.03), 0.003 |
| Trial*Age | -0.03 (0.01), -0.01 | -0.01 (0.01), -0.004 |
| Constant | **3.04^***^ (0.12)** | **2.27^***^ (0.11)** |

*Note*. All continuous predictors were scaled and centered. For each data point of the dependent variable, models used the most recent preceding data point for all trial-level independent variables (e.g., *Score* refers to the participants’ score on the snake arcade game of the previous trial in the model predicting point stealing and of the same trial in the model predicting rank buying). *Within-person mean rank* was included in the model predicting rank buying to account for the increase in rank resulting from previous rank buying choices. Legend: ^*^p<0.05; ^**^p<0.01; ^***^p<0.001.

**Supplemental Table S2**

*Main models predicting point stealing tested with other subdivisions of early- vs late-onset attempters*

| Dependent variable: | **Point stealing – prevention of one-on-one defeat [H1]**  B (standard error), standardized β | | | | |
| --- | --- | --- | --- | --- | --- |
| Cutoff for early- vs late-onset attempters: | **< 43 vs ≥ 43 years**  (median split) | | **< 60 vs ≥ 60 years**  (standard cutoff for old age) | |  |
| Models: | **Main Model 1a**  (testing group differences in the effect of trial) | **Main Model 1b**  (testing group differences in the effect of last trial’s outcome) | **Main Model 1a**  (testing group differences in the effect of trial) | **Main Model 1b**  (testing group differences in the effect of last trial’s outcome) |  |
| *Trial-level (design) variables* |  |  |  |  |  |
| Trial | **0.23*** (0.04), 0.13** | **0.12*** (0.01), 0.07** | **0.24*** (0.03), 0.13** | **0.12*** (0.01), 0.07** |  |
| Victory (vs Defeat) | -0.01 (0.03), -0.01 | -0.04 (0.07), -0.03 | -0.02 (0.03), -0.01 | -0.04 (0.06), -0.02 |  |
| Opponent’s rank | **0.09*** (0.01), 0.06** | **0.09*** (0.01), 0.06** | **0.09*** (0.01), 0.06** | **0.09*** (0.01), 0.06** |  |
| Score | -0.001 (0.02), -0.001 | 0.001 (0.02), 0.0005 | 0.001 (0.02), 0.0005 | 0.001 (0.02), 0.0005 |  |
| Current rank | **0.06* (0.02), 0.03** | **0.06* (0.02), 0.03** | **0.06* (0.02), 0.03** | **0.06* (0.02), 0.03** |  |
| *Study groups* |  |  |  |  |  |
| Study group (ref: Early-onset attempters): |  |  |  |  |  |
| Late-onset attempters | -0.08 (0.34), -0.05 | -0.09 (0.34), -0.05 | 0.15 (0.44), 0.07 | 0.15 (0.44), 0.09 |  |
| Depressed non-attempters | -0.02 (0.28), -0.02 | -0.07 (0.28), -0.04 | 0.06 (0.23), 0.03 | 0.01 (0.23), 0.01 |  |
| Non-psychiatric comparisons | -0.05 (0.31), -0.04 | -0.06 (0.31), -0.04 | 0.02 (0.27), 0.01 | 0.01 (0.27), 0.01 |  |
| Study group*Trial (ref: Early-onset attempters*Trial): |  |  |  |  |  |
| Late-onset attempters*Trial | **-0.13** (0.05), -0.07** | - | **-0.33*** (0.07), -0.19** | - |  |
| Depressed non-attempters*Trial | **-0.15*** (0.04), -0.08** | - | **-0.15*** (0.03), -0.08** | - |  |
| Non-psychiatric comparisons*Trial | **-0.15** (0.04), -0.08** | - | **-0.15*** (0.04), -0.08** | - |  |
| Study group*Victory (ref: Early-onset attempters*Victory): |  |  |  |  |  |
| Late-onset attempters*Victory | - | 0.01 (0.10), 0.01 | - | 0.02 (0.13), 0.01 |  |
| Depressed non-attempters*Victory | - | 0.12 (0.08), 0.07 | - | 0.12 (0.07), 0.07 |  |
| Non-psychiatric comparisons*Victory | - | 0.01 (0.09), 0.01 | - | 0.01 (0.08), 0.004 |  |
| *Participant-level covariates* |  |  |  |  |  |
| Male sex (vs female) | -0.01 (0.19), -0.003 | 0.02 (0.19), 0.01 | -0.02 (0.19), -0.01 | 0.01 (0.19), 0.005 |  |
| Age | -0.11 (0.09), -0.07 | -0.12 (0.08), -0.07 | -0.12 (0.10), -0.07 | -0.13 (0.10), -0.08 |  |
| Male sex*trial (vs female sex*Trial) | 0.02 (0.03), 0.01 | - | 0.01 (0.03), 0.01 | - |  |
| Age*Trial | -0.02 (0.01), -0.01 | - | -0.002 (0.01), -0.001 | - |  |
| Male sex*Victory (vs female sex*Victory) | - | -0.07 (0.05), -0.04 | - | -0.07 (0.05), -0.04 |  |
| Age*Victory | - | 0.03 (0.03), 0.02 | - | 0.03 (0.03), 0.02 |  |
| Constant | **3.07*** (0.24)** | **3.09*** (0.24)** | **3.00*** (0.20)** | **3.02*** (0.20)** |  |

*Note*. All continuous predictors were mean centered and scaled. For each data point of the dependent variable, models used the most recent preceding data point for all trial-level independent variables (e.g., *Score* refers to the participants’ score of the previous trial). Legend: *, p<0.05; **, p<0.01; ***, p<0.001.

**Supplemental Table S3**

*Main models predicting rank buying tested with other subdivisions of early- vs late-onset attempters*

| Dependent variable: | **Rank buying – prevention of loss of status [H2]**  B (standard error), standardized β | | | | | |
| --- | --- | --- | --- | --- | --- | --- |
| Cutoff for early- vs late-onset attempters: | **< 43 vs ≥ 43 years**  (median split) | | | **< 60 vs ≥ 60 years**  (standard cutoff for old age) | |  |
| Models: | **Main Model 1a**  (testing group differences in the effect of trial) | **Main Model 1b**  (testing group differences in the effect of last trial’s outcome) | **Main Model 1a**  (testing group differences in the effect of trial) | | **Main Model 1b**  (testing group differences in the effect of last trial’s outcome) |  |
| *Trial-level (design) variables* |  |  |  | |  |  |
| Trial | 0.06 (0.04), 0.04 | **0.07*** (0.01), 0.04** | -0.02 (0.06), -0.01 | | **0.07*** (0.01), 0.04** |  |
| Victory (vs Defeat) | **-0.06* (0.03), -0.03** | **-0.18* (0.08), -0.11** | **-0.06* (0.03), -0.03** | | **-0.45*** (0.12), -0.28** |  |
| Opponent’s rank | 0.03 (0.01), 0.02 | 0.03 (0.01), 0.02 | 0.03 (0.01), 0.02 | | 0.03 (0.01), 0.02 |  |
| Score | 0.001 (0.01), 0.001 | 0.002 (0.01), 0.001 | 0.002 (0.01), 0.001 | | 0.002 (0.01), 0.001 |  |
| Current rank | **0.07** (0.02), 0.05** | **0.08*** (0.02), 0.05** | **0.07** (0.02), 0.05** | | **0.08*** (0.02), 0.05** |  |
| Within-person mean rank buying | **0.18*** (0.01), 0.11** | **0.18*** (0.01), 0.11** | **0.18*** (0.01), 0.11** | | **0.18*** (0.01), 0.11** |  |
| *Study groups* |  |  |  | |  |  |
| Study group (ref: Late-onset attempters): |  |  |  | |  |  |
| Early-onset attempters | -0.03 (0.31), -0.01 | -0.11 (0.31), -0.07 | 0.07 (0.40), 0.05 | | -0.09 (0.40), -0.05 |  |
| Depressed non-attempters | -0.08 (0.25), -0.05 | -0.11 (0.25), -0.07 | -0.01 (0.37), -0.003 | | -0.13 (0.37), -0.08 |  |
| Non-psychiatric comparisons | 0.03 (0.27), 0.02 | -0.03 (0.28), -0.02 | 0.09 (0.39), 0.06 | | -0.04 (0.39), -0.03 |  |
| Study group*Trial (ref: Late-onset attempters*Trial): |  |  |  | |  |  |
| Early-onset attempters*Trial | 0.04 (0.05), 0.03 | - | **0.13* (0.06), 0.08** | | - |  |
| Depressed non-attempters*Trial | -0.02 (0.04), -0.01 | - | 0.06 (0.06), 0.04 | | - |  |
| Non-psychiatric comparisons*Trial | 0.01 (0.04), 0.01 | - | 0.10 (0.06), 0.06 | | - |  |
| Study group*Victory (ref: Late-onset attempters*Victory): |  |  |  | |  |  |
| Early-onset attempters*Victory | - | **0.27** (0.10), 0.17** | - | | **0.52*** (0.13), 0.32** |  |
| Depressed non-attempters*Victory | - | 0.10 (0.08), 0.06 | - | | **0.38** (0.12), 0.23** |  |
| Non-psychiatric comparisons*Victory | - | 0.17 (0.09), 0.11 | - | | **0.45*** (0.13), 0.27** |  |
| *Participant-level covariates* |  |  |  | |  |  |
| Male sex (vs female) | 0.23 (0.17), 0.14 | 0.23 (0.17), 0.14 | 0.23 (0.17), 0.14 | | 0.24 (0.17), 0.14 |  |
| Age | -0.07 (0.08), -0.04 | -0.08 (0.08), -0.05 | -0.06 (0.09), -0.04 | | -0.09 (0.09), -0.05 |  |
| Male sex*trial (vs female sex*Trial) | 0.01 (0.03), 0.01 | - | 0.01 (0.03), 0.01 | | - |  |
| Age*Trial | -0.01 (0.01), -0.004 | - | 0.002 (0.01), 0.001 | | - |  |
| Male sex*Victory (vs female sex*Victory) | - | 0.003 (0.06), 0.002 | - | | -0.02 (0.05), -0.01 |  |
| Age*Victory | - | 0.05 (0.03), 0.03 | - | | **0.08** (0.03), 0.05** |  |
| Constant | **2.30*** (0.23)** | **2.34*** (0.23)** | **2.23*** (0.36)** | | **2.36*** (0.36)** |  |

*Note*. All continuous predictors were mean centered and scaled. For each data point of the dependent variable, models used the most recent preceding data point for all trial-level independent variables (e.g., *Score* refers to the participants’ score of the same trial). *Within-person mean rank* was included in the models to account for the increase in rank resulting from previous rank buying choices. Legend: *, p<0.05; **, p<0.01; ***, p<0.001.
